# Supplementary material for: Link between supercurrent diode and anomalous Josephson effect revealed by gate-controlled interferometry
Source: Nat Commun. 2024 May 23;15:4413. doi: 10.1038/s41467-024-48741-z (PMC11116472; doi:10.1038/s41467-024-48741-z)
Supplement: Supplementary file 1 — Supplementary Information [file 41467_2024_48741_MOESM1_ESM.pdf]

Supplementary Information: Link between supercurrent diode and anomalous Josephson effect revealed by gate-controlled interferometry

S. Reinhardt, T. Ascherl, J. Berger, N. Paradiso,* and C. Strunk
Institut für Experimentelle und Angewandte Physik,
University of Regensburg, 93040 Regensburg, Germany

A. Costa and J. Fabian
Institut für Theoretische Physik, University of Regensburg, 93040 Regensburg, Germany

D. Kochan
Institute of Physics, Slovak Academy of Sciences, 84511 Bratislava, Slovakia
Center of Quantum Frontiers of Research and Technology (QFort),
National Cheng Kung University, Tainan 70101, Taiwan and
Institut für Theoretische Physik, University of Regensburg, 93040 Regensburg, Germany

S. Gronin and G. C. Gardner
Birck Nanotechnology Center, Purdue University, West Lafayette, Indiana 47907 USA

T. Lindemann
Birck Nanotechnology Center, Purdue University, West Lafayette, Indiana 47907 USA and
Department of Physics and Astronomy, Purdue University, West Lafayette, Indiana 47907 USA

M. J. Manfra
Birck Nanotechnology Center, Purdue University, West Lafayette, Indiana 47907 USA
Department of Physics and Astronomy, Purdue University, West Lafayette, Indiana 47907 USA
School of Materials Engineering, Purdue University, West Lafayette, Indiana 47907 USA and
Elmore Family School of Electrical and Computer Engineering,
Purdue University, West Lafayette, Indiana 47907 USA

WAFER GROWTH AND SAMPLE FABRICATION

The hybrid superconductor-semiconductor layer stack is shown in Fig. S1**a**. The active layer consists of a bottom barrier of 4 nm $In_{0.75}Ga_{0.25}As$, a 7 nm InAs quantum well, and a top barrier of 10 nm $In_{0.75}Ga_{0.25}As$. Growth of the semiconductor stack is followed by in-situ deposition of 5 nm aluminum [1].

The mesa of the SQUID device is wet etched using a solution of H_3PO_4:$C_6H_8O_7$:H_2O_2:H_2O (1.2:14:2:88) to a depth of 250 nm. The Josephson junctions are defined using standard electron beam lithography. Prior to etching of the junctions, we expose the developed structures to oxygen plasma for 5 s in order to remove residues of PMMA resist. Selective etching of the aluminum in the junctions is then performed with aluminum etchant type D from Transene at 50 °C for 3 s. Both junctions are covered with 60 nm aluminum oxide grown by atomic layer deposition at 80 °C. Before the first pulse (trimethylaluminum), the sample is dried under nitrogen flow for 2 hours. Finally, a Ti/Au (5 nm / 100 nm) top-gate is deposited onto JJ2 by electron beam evaporation.

BASIC CHARACTERIZATION OF THE QUANTUM WELL AND THE SUPERCONDUCTOR

Basic transport properties of the semiconductor quantum well are performed with a standard Hall bar device. The aluminum film is removed from the Hall bar and a top-gate is fabricated using the methods described above. In Figures S1**b-e** we show Hall mobility, mean free path, Fermi wavelength, and Fermi velocity as a function of carrier density at $T = 1.5$ K. The peak mobility $\mu > 60000$ cm^2/Vs is obtained at the density $n \sim 5 \times 10^{11}$ cm^{-2}.

The electron effective mass is determined from the temperature dependence of Shubnikov-de Haas (SdH) oscillations, following [2]. Figure S1**f** shows SdH oscillations in the resistance with a second-order polynomial background removed in order to obtain the amplitude of each oscillation as a function of temperature. The carrier density obtained from the oscillations is $\sim 7 \times 10^{11}$ cm^{-2}. For each maximum and minimum of the oscillations we obtain an effective mass

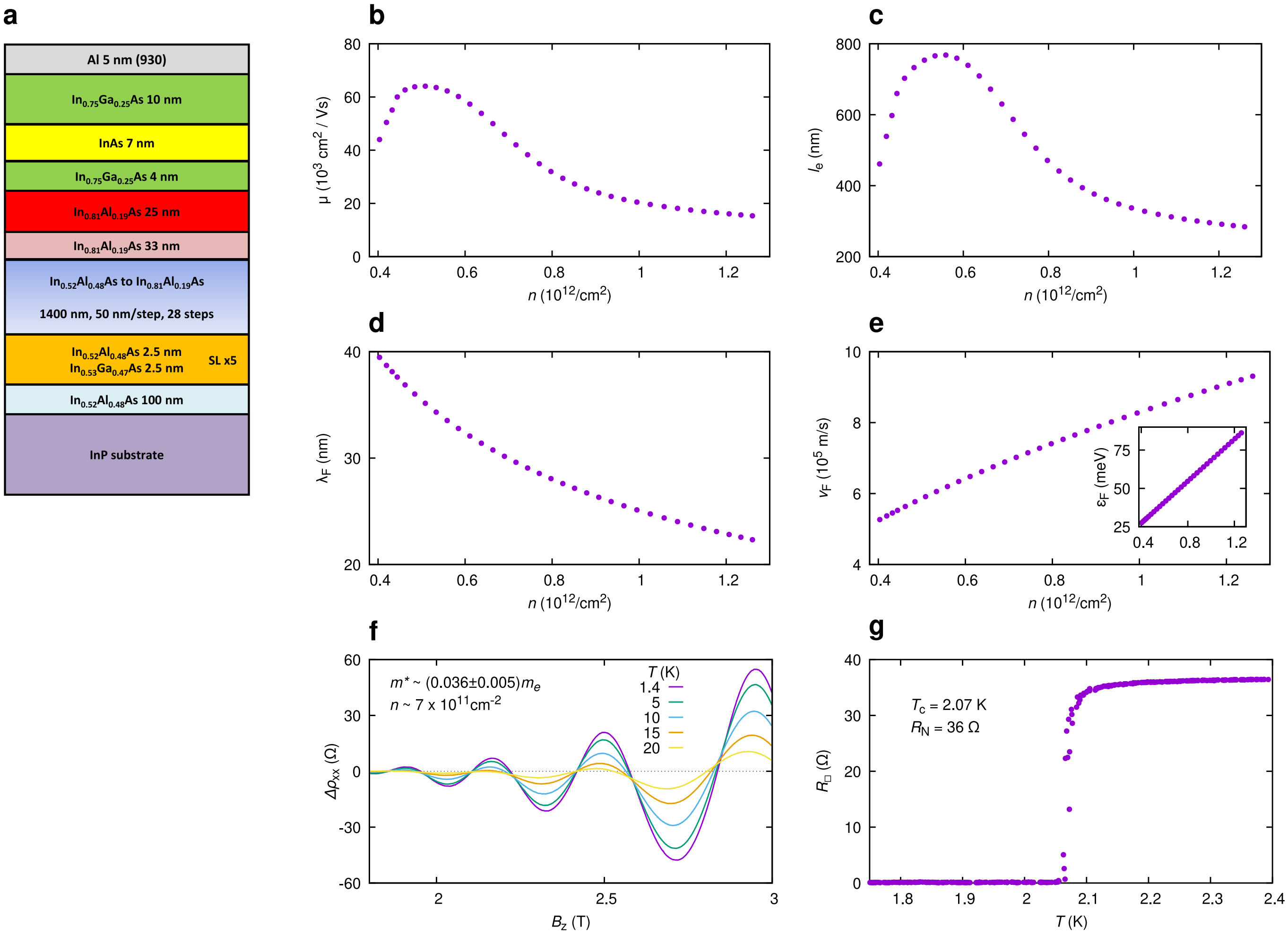

FIG. S1. **a**, Layer stack of the used wafer. **b-e**, Transport properties of the quantum well measured with a top gated Hall bar at temperature $T = 1.5$ K. The inset of panel **e** shows the Fermi level ε_F versus n. The range in gate voltage corresponding to the n-range displayed in panels **b-e** is [-3.5 V,-0.2 V]. **f**, Amplitude of Shubnikov-de Haas oscillations for temperatures between 1.4 K and 20 K. **g** $R(T)$ Characteristic of a Hall bar device with the pristine aluminum film.

by fitting the temperature dependence of the amplitude with the formula

$$A_{\mathrm{SdH}}(T) \sim \frac{2\pi^2 k_B T/\hbar\omega_c}{\sinh(2\pi^2 k_B T/\hbar\omega_c)} \tag{S.1}$$

The obtained mass $m^*/m_e = 0.036 \pm 0.005$ is slightly lower compared to the value $m^*/m_e = 0.04$ found in [2].

Figures S1**g** shows the resistance of the heterostructure, including the aluminum film, as a function of temperature, measured in a Hall bar geometry. Due to the low thickness of the film, the critical temperature is enhanced to 2.07 K with a normal state sheet resistance of 36 Ω.

MEASUREMENT TECHNIQUES AND DATA EVALUATION

Measurements are performed in a dilution cryostat with a base temperature of 40 mK. Differential resistance $dV/dI(I_{DC})$ is measured in a standard 4-point setup using a current excitation of $I_{\mathrm{ac}} = 10$ nA(rms) at frequency $f = 2.333$ kHz. The voltage is measured using a differential bipolar junction preamplifier (SR552) and a digital lock-in amplifier (SR830). All measurement lines are filtered at the head of the cryostat using LC filters (Tusonix 4201-053). Below the mixing chamber we use highly resistive CuNi/CuNi coaxial lines (GVLZ185) with a large attenuation above 100 MHz. The fields B_z and B_y are applied using a 2-axis vector magnet, and the rotation of the in-plane field is

performed by mounting the sample on an Attocube piezo rotator (ANRv220). A resolution below 50 nT in B_z is realized by exchanging the magnet power supply with a high precision current source (Keysight B2961B). Before starting a measurement at a new configuration of in-plane field and/or rotation angle we heat our sample above the critical temperature $T_c \sim 2K$ and perform a field cooling in zero out-of-plane field. The gate voltage V_g is applied using a source meter (Keysight B2901B). For gate voltages in the range $V_g = -2.5$ V... 1 V the leakage current is below the resolution of the instrument ($|I_{\mathrm{leak}}| < 1$ pA). For the definition of the SQUID critical current we use a threshold resistivity of $dV/dI = 6\ \Omega$. Using a different definition for the threshold has no visible effect on the results.

For the determination of φ_0 and η the SQUID critical current $I_c(B_z)$ is measured for the sequence of gate voltages $V_g = -2.5$ V, -1.5 V, ..., 0.5 V, 1 V. The accuracy is improved by repeatedly cycling the gate voltage through this sequence (40 cycles for most measurements). After obtaining φ_0 and η we average over all cycles. The error bars in Fig. 2**e** for the values of η are found as the standard error of the mean.

MODELING OF INDUCTIVE SCREENING EFFECTS IN THE ASYMMETRIC SQUID

The obtained critical currents of the SQUID device are strongly affected by the large kinetic inductance of the aluminum electrodes. A minimal circuit model of our device, which includes the inductance of the electrodes of JJ1 and the loop, is show in Figure S2**a**. The reference junction JJ1 is modeled as a network of N JJs in parallel, each with critical current $I_{c,1}/N$. Setting $N = 20$ is sufficient to model the central lobe of the Fraunhofer interference pattern of JJ1. The effective area of JJ1 is A_{ref}, while the area of the large loop is A_{loop}. The positive and negative critical currents for a given out-of-plane field B_z can be calculated as a function of the phase difference γ_{JJ2} over JJ2. Given γ_{JJ2} the phase difference $\gamma_{\mathrm{JJ1,N}}$ over the rightmost junction in JJ1 is

$$\gamma_{\mathrm{JJ1},N} = \gamma_{\mathrm{JJ2}} - 2\pi(B_z A_{\mathrm{loop}} - L_{\mathrm{loop}} I_{c,2} \sin(\gamma_{\mathrm{JJ2}}))/\Phi_0 \tag{S.2}$$

The phase differences $\gamma_{\mathrm{JJ1},i}$ are found by iteration:

$$\gamma_{\mathrm{JJ1},i} = \gamma_{\mathrm{JJ1},i+1} - 2\pi(B_z A_{\mathrm{ref}}/(N-1) - L_{\mathrm{ref}}/(N-1) I_i)/\Phi_0 \tag{S.3}$$

where $I_i = I_{i+1} + I_{c,1}/N \sin(\gamma_{\mathrm{JJ1},i+1})$ is the current flowing through the inductor between junctions i and $i+1$. The positive and negative critical currents are then found as the extremal values of $I_0(\gamma_{\mathrm{JJ2}})$. In FiguresS2**b-d** we model the SQUID critical current obtained for a in-plane-field $|B_y| = 100$ mT. A good match to the data can be found with the following parameters: $A_{\mathrm{loop}} = 1150$ (µm)^2, $I_{c,1} = 2.48\ \mu$A, $A_{\mathrm{ref}} = 70$ (µm)^2, $L_{\mathrm{ref}} = 0.8$ nH, and $L_{\mathrm{loop}} = 1.5$ nH. From the value of L_{loop} we determine the sheet inductance of the aluminum film $L_\square = L_{\mathrm{loop}}/N_{\square,\mathrm{loop}} = 1.5$ nH/46 ~ 30 pH. The kinetic inductance of the loop largely exceeds the geometric inductance which we estimate as $L_{\mathrm{loop,geo}} \lesssim 100$ pH.

The amplitude of the SQUID oscillations at $V_g = 1$ V, $By = -100$ mT and $T = 40$ mK is matched by setting $I_{c,2} = 400$ nA. Figure S2**b** shows the SQUID critical current for different values of $I_{c,2}$ near the maximum of the SQUID critical current. Importantly, the crossing point with the reference line ($I_{c,2} = 0$) is the same for all values of $I_{c,2}$, showing that screening has no effect on our evaluation of $\Delta\varphi_0$.

Figure S2**c** shows the results of SQUID oscillation measurements performed for $V_g = 1.0$ V (green) and $V_g = -2.5$ V (purple), at $B_y = -100$ mT and $T = 40$ mK. A comparison with the results of our calculations in Fig. S2**d** makes it evident that our simple model described above correctly captures the behavior of the SQUID. We want to stress that our asymmetric SQUID displays a trivial supercurrent diode behavior (in Fig. S2**c,d** in general $I_c^+(B_z) \neq I_c^-(B_z)$). This is a long known feature of asymmetric SQUIDs and it is not the focus of our work. Instead, our focus is on the physics of a single, homogeneous Josephson junction (JJ2).

From the analysis of the inductive screening effects discussed above, we can conclude that:

- Owing to screening effects, the SQUID oscillations do not reproduce the CPR of JJ2. However, for positive bias, one can still deduce $I_{c,2}^+$ from the maximum of the oscillations, since these two quantities are proportional, see Fig. S2**e,f**. Instead, **for positive bias, one cannot deduce $I_{c,2}^-$ from the minima of the SQUID oscillations**. The correct way to deduce $I_{c,2}^-$ is to look at oscillations for negative current bias. $I_{c,2}^-$ is proportional to the **minima** of the SQUID oscillations for **negative bias**, see Fig. 2**c** of the main text.

- As it is clearly visible in Figures S2**c,d**, the amplitudes of the fast SQUID oscillations are not constant. This is caused by a coupling of the loop current I_{JJ2} to the flux of JJ1. The flux in JJ1 is changed by $\Phi \sim L_{\mathrm{ref}} I_{\mathrm{JJ2}}$, modulating the critical current of JJ1. For the measurement of critical currents, demonstrated in Figure 2 of the main text, we find that the critical currents for different gate voltages are only proportional to $I_{c,2}(V_g)$,

with the proportionality constant depending on the chosen SQUID oscillation (i.e., on the exact B_z value). As positive and negative critical currents need to be measured at opposite current bias (see previous points), the slightly different scale factors result in offsets for the extracted diode efficiency $\eta = 2(I_c^+ - |I_c^-|)/(I_c^+ + |I_c^-|)$. We note that the gate slope $d\eta/dV_g$ is nevertheless a well defined quantity and not affected by the screening effects.

- As a consequence of the B_z-dependence of the amplitude of the SQUID oscillations, the correct way to compare an oscillation with positive bias with one with negative bias is to consider oscillations with opposite B_z, namely oscillations which are inversion-symmetric around the origin of the $I_c(B_z)$ graph, see arrows in Fig. 2**a** of the main text. Indeed, by the global time-reversal symmetry $I_c^+(B_z) = -I_c^-(-B_z)$ in absence of in-plane field. Taking precisely opposite oscillations, as described in the previous point, should make screening effect irrelevant, so that, in principle, the observation of finite η for finite $B_{\rm ip}$ should be considered as a real effect and not an artifact of screening. However, the combination of φ_0-shift and the strong dependence of the SQUID oscillations on B_z makes it possible to measure (following the aforementioned procedure) a finite η even in absence of a genuine SDE in JJ2. In fact, owing to the finite φ_0, opposite SQUID oscillations do not occur anymore at exactly opposite B_z, and the strong B_z dependence of the SQUID oscillations can render the opposite oscillation amplitudes not exactly equal even in absence of intrinsic SDE. The effect is very small, still we maintained a conservative approach and decided to look at $\partial\eta/\partial V_g$ instead of η itself. Also, we can exclude that the gate dependence of η is a simple artifact of that of φ_0 (due to the mechanism just described) for the following reasons: (i) from our calculations the measured η is too large to be entirely generated by the mechanism just described (φ_0 plus B_z-dependent oscillations). (ii) The gate dependence of φ_0 and η are different: the former is nonlinear, the latter is linear (compare Fig. 1**g** and Fig. 2**e** of the main text). (iii) The temperature dependence of $\Delta\varphi_0$ and that of $\partial\eta/\partial V_g$ is dramatically different, see Fig. 3 of the main text.

- From Fig. S2**b** we can conclude that inductive screening does not affect the value of B_z at which $I_2 = 0$ (the curves all cross the baseline at the same point). Clearly, for positive bias, it is important to only consider the crossing with the baseline *with positive slope*, which is the significant one. This explains our operational definition of B_0, which is important to determine φ_0.

- The inductive screening effects depend on the kinetic inductance of the Al film and on the magnitude of the supercurrent flowing the in the SQUID arms. By increasing $B_{\rm ip}$ in the range under study, the decrease of the critical current I_c is more important than the increase of the kinetic inductance of the arms. Therefore, the larger $B_{\rm ip}$, the smaller the impact of screening. At fields $B_{\rm ip} < 100$ mT the screening effects prevent the determination of the anomalous phase shift, as the $I_c(B_z)$ curves no longer intersect the baseline. An example of this can be seen in Figures 2**b**,**c** of the main text, where $B_{\rm ip} = 50$ mT.

We have also performed simulations with non-sinusoidal current phase relations of JJ2. This has no implications for the extraction of $\Delta\varphi_0$ and the diode efficiency. In Figure S2**g** we show a simulation where we use the fully ballistic current phase relation for JJ2:

$$I_2(\gamma) = \frac{I_{c,2}\sin(\gamma)}{2\sqrt{1-\sin^2(\gamma/2)}} \tag{S.4}$$

For this case we also find the proportionality between extracted critical current and real critical current, as shown in Figure S2**h**.

GATE DEPENDENCE OF THE MAGNETOCHIRAL ANISOTROPY FOR THE INDUCTANCE

In this section we comment about the gate dependence of the magnetochiral anisotropy (MCA) of the inductance, which we measured in our previous work, Ref. [3]. In that work, we established an analogy between the known MCA for the resistance (e.g. in the fluctuation regime of a noncentrosymmetric superconductor) and MCA for the inductance. This latter was the new quantity introduced in Ref. [3], where we showed that the Josephson inductance can be written as

$$L = L_0[1 + \gamma_L \hat{e}_z \cdot (\vec{B} \times \vec{I})], \tag{S.5}$$

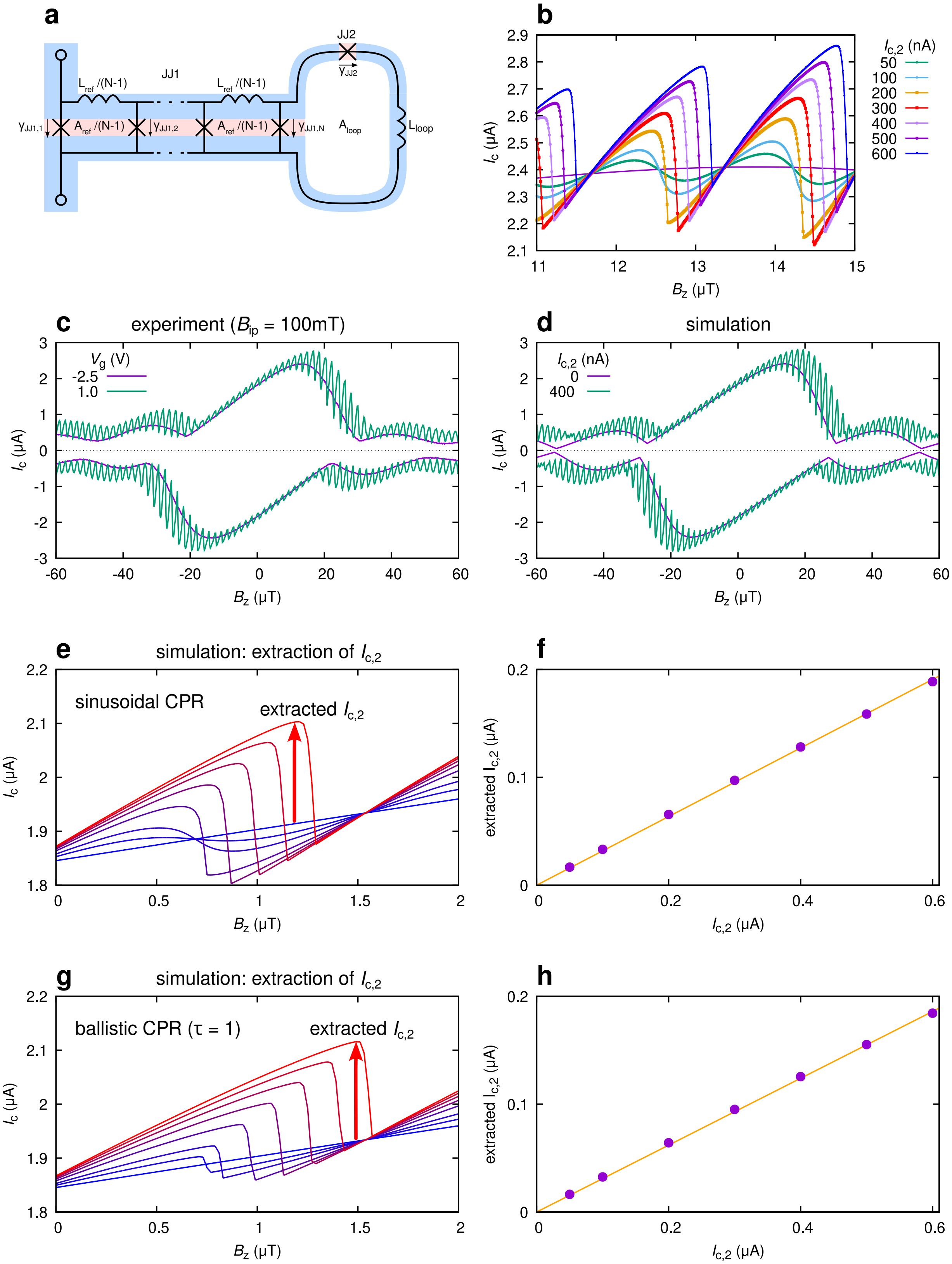

FIG. S2. **a**, Minimal circuit model of the SQUID device. **b,d**, Simulated critical currents of the SQUID with parameters as described in the text. **c**, experimental values of positive and negative critical currents for $B_{\mathrm{ip}} = 100$ mT and $\theta = -90$ °. **e**, Extraction of critical current $I_{c,2}$ from the SQUID oscillations. The CPR of JJ2 is $I_2(\gamma) = I_{c,2}\sin(\gamma)$. **f**, Dependence between extracted and actual critical current with linear fit **g**, Extraction of the critical current for the case where JJ2 has a fully ballistic CPR. **h** Dependence between extracted and actual critical current for the case of a ballistic CPR with linear fit.

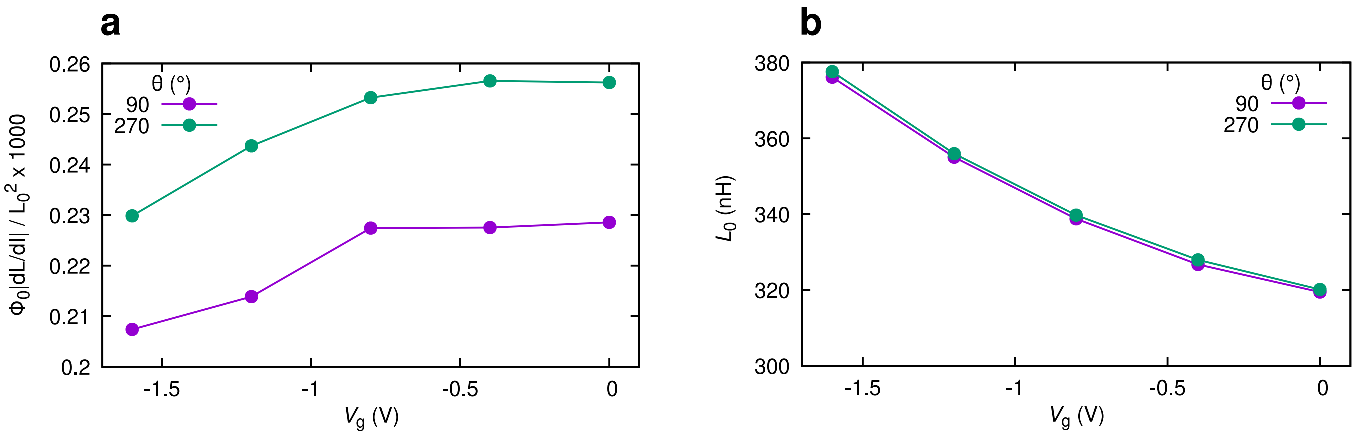

FIG. S3. Magnetochiral anisotropy of the Josephson inductance for 1D array at $B_{\rm ip} = 100$ mT.

where $\gamma_L \equiv L_0'/(L_0 B_{ip})$, with $L_0' \equiv \frac{dL}{dI}|_{I=0}$. Equation S.5 mirrors the Rikken formula [4]

$$R = R_0[1 + \gamma \hat{e}_z \cdot (\vec{B} \times \vec{I})], \tag{S.6}$$

which holds for noncentrosymmetric conductors with nonreciprocal resistance, for which γ is defined as $R_0'/(R_0 B_{ip})$, with $R_0' \equiv \frac{dR}{dI}|_{I=0}$.

It must be kept in mind that both the MCA coefficient for the resistance (γ) and that for the inductance (γ_L) are dimensional quantities which scale with the inverse of the sample width in quasi 2D conductors. In Ref. [3], we have plotted in Fig. 2d the gate dependence γ_L. At first glance, it might appear that no dependence is observed (the curves in Fig. 2d of Ref. [3] fall on top of each other). However, it must be kept in mind that γ_L trivially depends on the number of channels N (or, equivalently, on the width). Since $I \propto N$ and $L_0 \propto N^{-1}$, then γ_L must scale as $1/N$. Therefore, in Ref. [3] the fact that γ_L appears constant is due to the fact that the expected increase of the specific magnetochiral character (considered "per channel") is compensated by the decrease due to the trivial increase of N (decrease of N^{-1}).

The best way to represent the gate-induced enhancement of the MCA for the inductance (which mirrors the increase in $|\varphi_0|$ and η with V_g demonstrated in the present article) is to represent the MCA with a quantity which does not depend on N. To this end, we plot in Fig. S3**a** the V_g dependence of the quantity $\Phi_0|L_0'|/L_0^2$ for the same data set of Fig. 2d in Ref. [3]. Data refer to $B_{\rm ip} = 100$ mT and $\theta = 90°$ (purple) and $\theta = 270°$ (green). We clearly observe an increase in the magnetochiral character with V_g. The increase is relatively small due to the fact that in that sample the gate had a much weaker effect compared to the sample studied in the present article. To give an idea of this, in Fig. S3**b** we plot L_0 as a function of V_g, showing that sweeping V_g from -1.5 to 0 V corresponds to a change in L_0 of less than 20%.

ANGLE DEPENDENCE OF THE ANOMALOUS PHASE SHIFT

Figure S4**a** shows the dependence of the anomalous phase difference $\Delta\varphi_0(V_g = 1.5\ \text{V}, B_{\rm ip} = 100\ \text{mT})$ on the rotation angle θ defined in the main text. The observed behaviour is in good agreement with the expected relation $\varphi_0 \sim \sin(\theta)$. In Figure S4**b** we plot $\Delta\varphi_0$ as a function of the B_y component of the in-plane field ($B_y := B_{ip}\sin(\theta)$), showing a good agreement with the expected linear behaviour.

EXTRACTION OF THE ANOMALOUS PHASE AT DIFFERENT SQUID OSCILLATIONS

In principle, every oscillation of the SQUID pattern can be used to obtain the anomalous phase shift $\Delta\varphi_0$. In praxis we use oscillations near the absulte maximum of the critical current, as there the amplitude of the oscillations is large. In Figure S5**a** we obtain the anomalous phase shift at three different SQUID oscillations. The extracted phase shifts from the different oscillations are nearly equal (Figure S5**b**).

So far, we have shown how $\Delta\varphi$ is determined from measurements of the positive critical current $I_c(B_z)$. It is also possible to obtain $\Delta\varphi$ from measurements of the negative critical current. Figure S5**a** shows data of the negative

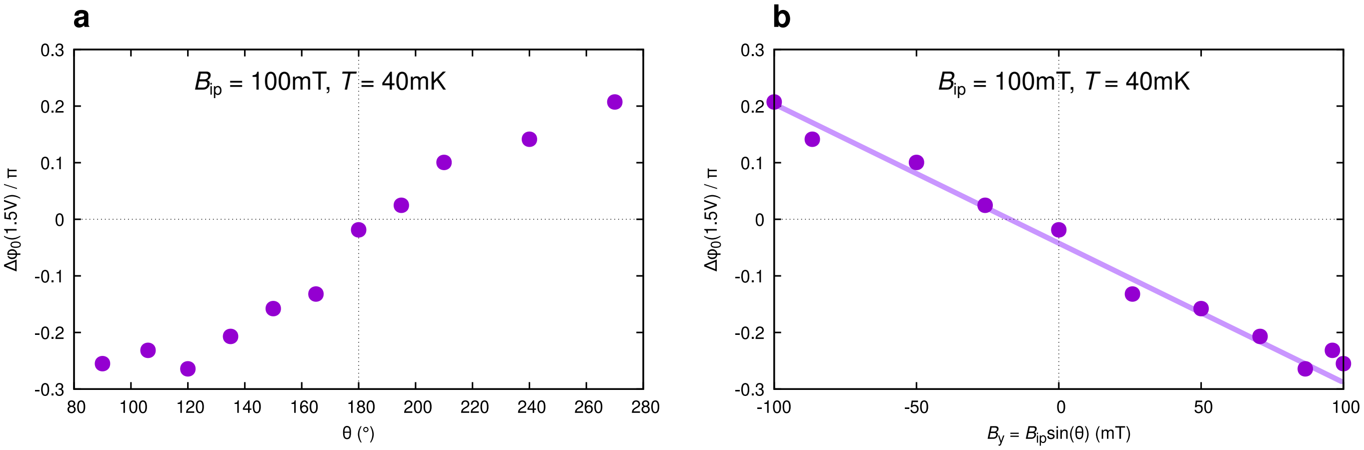

FIG. S4. **a**, Anomalous phase shift for $B_{ip} = 100$ mT as a function of θ as defined in the main text. **b**, Anomalous phase shift as a function of $B_y = B_{ip}\sin(\theta)$.

critical current $I_c(B_z)$ obtained at $B_{\mathrm{ip}} = 100$ mT and $\theta = -90$ °. The extracted anomalous phase shift is shown in Figure S5**b** and is in good agreement with the phase shift extracted from positive critical currents (see Figure 1**d** of the main text).

SUPPLEMENTAL DATA ON TEMPERATURE DEPENDENCE

Figure S6**a** shows the temperature dependence of the differential resistance dV/dI of the SQUID for zero DC bias current and zero magnetic field. The positive and negative critical currents $I^{\pm}_{c,2}$ of JJ2 corresponding to Fig. 3**c** and **d** of the main text are shown in Fig. S6**b**. At $T = 750$ mK the critical current is reduced by ~ 50 %. Fig. S6**c** and **d** show $\eta(V_g)$ for the temperatures $T = 40$ mK and $T = 750$ mK (corresponding to the first and last data points in Fig. 3**d** of the main text). At $T = 750$ mK we observe larger statistical errors in the determination of the critical currents, leading to the larger error bars. By increasing the temperature, the superconducting-to-normal transition in the differential resistance becomes less sharp, leading to the increased noise in the determination of critical currents, and thereby the diode efficiency.

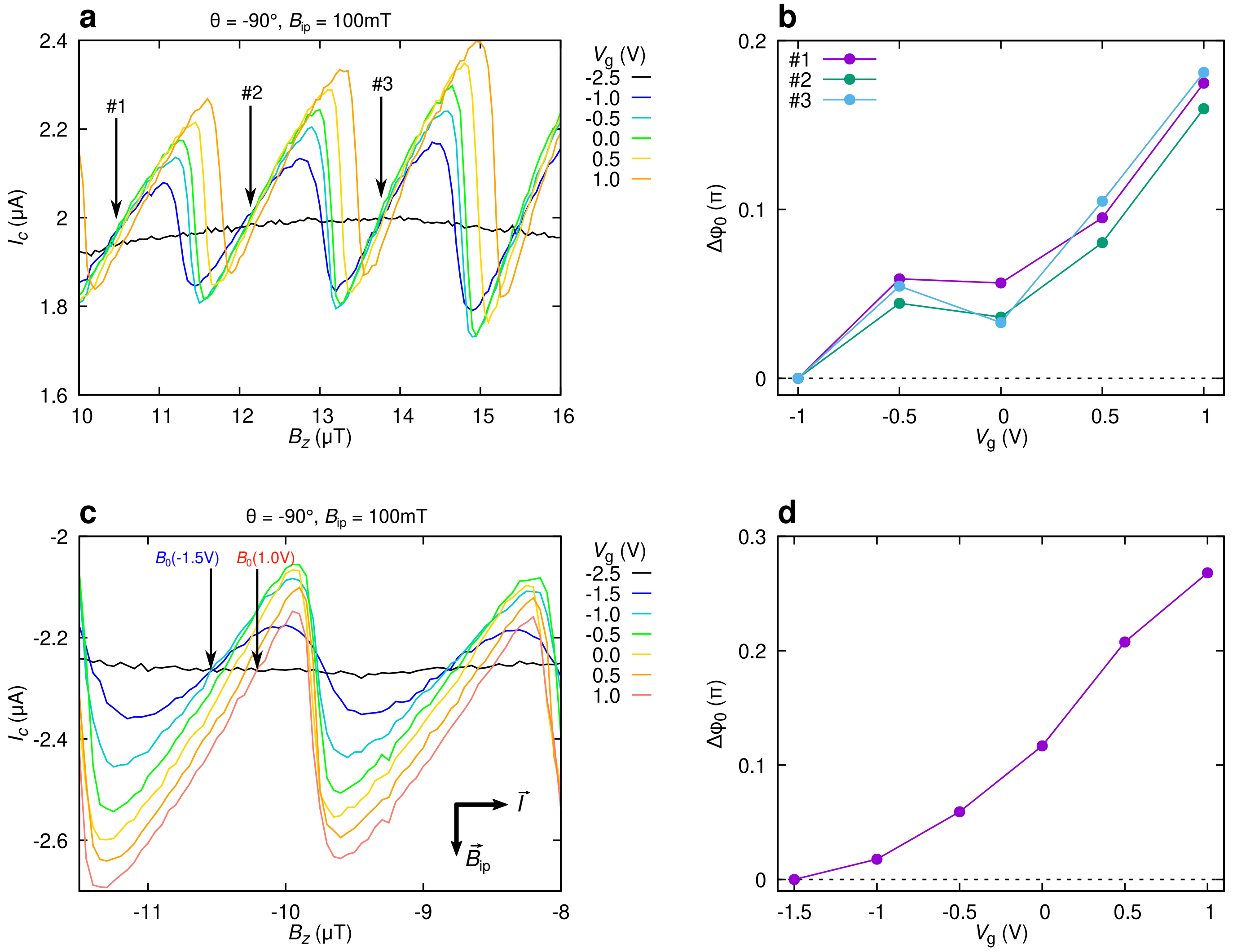

FIG. S5. **a**, SQUID critical current measured for multiple SQUID oscillations. The arrows mark the intersection with the baseline curve ($V_g = -2.5$ V) for three different oscillations. **b**, Anomalous phase shift $\Delta\varphi_0$ obtained at the different SQUID oscillations shown in panel **a**. **c**, SQUID critical current measured at negative bias current. All parameters are as for the measurement shown in Figure 1**d** of the main text. **d**, Anomalous phase shift $\Delta\varphi_0$ extracted from the measurement at negative bias current.

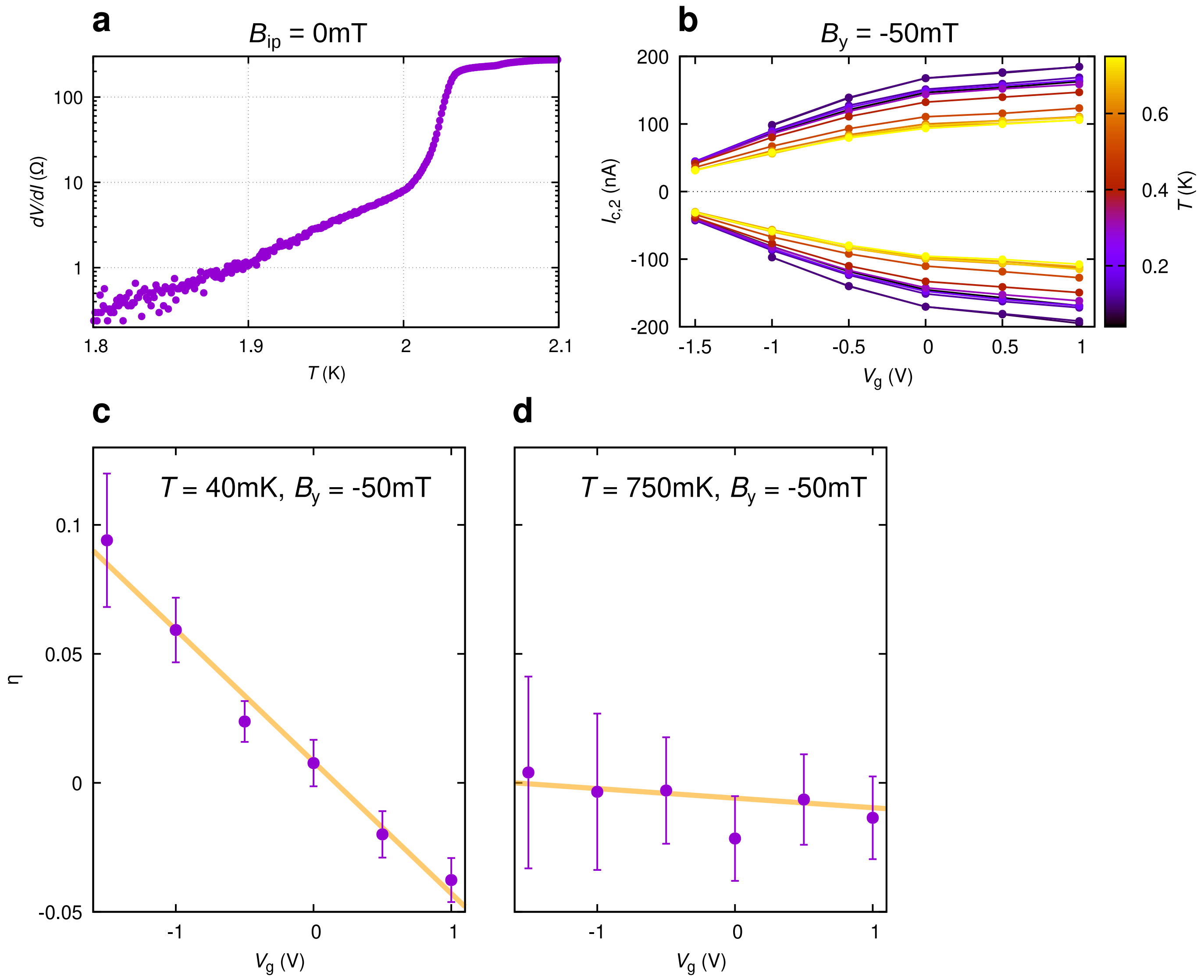

FIG. S6. **a**, $dV/dI(T)$ of the SQUID device at zero DC bias and zero magnetic field. **b**, Critical current $I^{\pm}_{c,2}(V_g)$ at $B_y = -50$ mT for different temperatures. **c** and **d**, Diode efficiency $\eta(V_g)$ for $T = 40$ mK and $T = 750$ mK.

* nicola.paradiso@physik.uni-regensburg.de

[1] T. Zhang, T. Lindemann, G. C. Gardner, S. Gronin, T. Wu, and M. J. Manfra, Mobility exceeding 100 000 cm^2/V s in modulation-doped shallow InAs quantum wells coupled to epitaxial aluminum, Phys. Rev. Mater. **7**, 056201 (2023).
[2] J. Yuan, M. Hatefipour, B. A. Magill, W. Mayer, M. C. Dartiailh, K. Sardashti, K. S. Wickramasinghe, G. A. Khodaparast, Y. H. Matsuda, Y. Kohama, Z. Yang, S. Thapa, C. J. Stanton, and J. Shabani, Experimental measurements of effective mass in near-surface inas quantum wells, Phys. Rev. B **101**, 205310 (2020).
[3] C. Baumgartner, L. Fuchs, A. Costa, S. Reinhardt, S. Gronin, G. C. Gardner, T. Lindemann, M. J. Manfra, P. E. Faria Junior, D. Kochan, J. Fabian, N. Paradiso, and C. Strunk, Supercurrent rectification and magnetochiral effects in symmetric Josephson junctions, Nature Nanotechnology **17**, 39 (2022).
[4] G. L. J. A. Rikken, J. Fölling, and P. Wyder, Electrical Magnetochiral Anisotropy, Phys. Rev. Lett. **87**, 236602 (2001).
